# The efficacy of polyether‐ether‐ketone wire as a retainer following orthodontic treatment

**DOI:** 10.1002/cre2.377

**Published:** 2020-12-13

**Authors:** Ammar Salim Kadhum, Akram Faisal Alhuwaizi

**Affiliations:** ^1^ Orthodontic Department, College of Dentistry University of Baghdad Baghdad Iraq

**Keywords:** PEEK, Retainer, Retention

## Abstract

**Objectives:**

To investigate the efficacy of polyether‐ether‐ketone (PEEK) wire as a fixed orthodontic retainer, by comparing its performance to other retainer wires and optimizing its adhesion to composite bonding materials.

**Materials and methods:**

Retainer wires of 15 mm segments were used, PEEK wires were prepared in cylindrical form with 0.8 mm diameter, and had two surface treatments namely air‐abrasion and conditioning with adhesive system. Three different metallic retainer wires were used for comparison and three tests were performed; two tests measured debonding force and associated wire deflection from acrylic blocks and bovine teeth and one test for pull‐out force. To test debonding force, a vertically directed compressive force was applied to the retainer wires bonded to the acrylic blocks and bovine teeth, while for pull‐out test; a vertically directed tensile force detached the retainer wire.

**Results:**

In both debonding tests, PEEK wires (regardless the surface treatment) had non‐significant difference when compared to each other, or to the other metallic wires, except the dead‐soft coaxial wire group. The dead‐soft coaxial wire group had significant difference when compared to other groups regarding both the force magnitude and maximum deflection, the only exception was the debonding force of the flat braided retainer wires bonded to bovine teeth. In pull‐out test PEEK wires conditioned with adhesive system and the air‐abraded recorded the second and third highest readings respectively.

**Conclusions:**

Within the limitations of this study, the 0.8 mm round PEEK wires have comparable performance—in terms of debonding and pull out forces—to conventional retainers when bonded with 4 mm composite bonding spots; using air‐abrasion for 10 s at 3.5 MPa provided sufficient adhesion of the composite to the wire, and conditioning with adhesive system may provide no further clinical benefit.

## 1 INTRODUCTION

Retention is an integral step of orthodontic treatment; it involves holding the teeth in their post‐treatment position, so that excellent long‐term result is obtained. Whenever intra‐arch instability is suspected, prolonged retention must be considered (Proffit et al., 2019). The long survival time of fixed retainers is one of the reasons that makes them the first choice for prolonged retention (Jin et al., 2018). Failure of bonded retainers, may occur in one of three sites, the wire composite interface, the adhesive‐enamel interface, and the wire itself. Failures at the first site are mostly related to composite (Bearn, 1995); failures at the second site are mostly attributed to moisture contamination or retainer wire movement during bonding (Butler & Dowling, 2005); lastly, failures at the third site are the result of stress‐fracture and are mostly associated with small diameter wires (Shaughnessy et al., 2016), and long service time (Lumsden et al., 1999). Failures will lead to changes in post treatment tooth position, that is, failed retention, either toward the pre‐treatment position, i.e. relapse, or to a totally new position (Butler & Dowling, 2005). However, failure of retention does occur sometimes without involving retainer failure (Sifakakis et al., 2015). This failure is the result of the development of force components within the wire as a result of improper contouring and adaptation at the time of bonding, or deformation developed due to masticatory forces for example (Shaughnessy et al., 2016; Sifakakis et al., 2011). Retention failure is not limited to a single type of fixed retainer, it can be seen with small diameter dead‐soft wires, small diameter flexible spiral wires, large diameter stiffer wires, and fiber‐reinforced composite (Sfondrini et al., 2020; Shaughnessy et al., 2016).

PEEK is attracting attention as an alternative to metal alloys in the dental field (Tada et al., 2017). It is a high‐temperature semi‐crystalline, and thermoplastic polymer, that is known for outstanding mechanical properties (Kurtz, 2019). PEEK shows lower deformations and higher fracture loads when processed by computer‐aided design/computer‐aided manufacturing (CAD/CAM) than can be achieved by other processes (Stawarczyk, Eichberger, et al., 2015). Good bonding to adhesives is an important requirement for a retainer wire (Annousaki et al., 2017). PEEK has known issues with bonding due to its inert behavior (Stawarczyk, Thrun, et al., 2015); therefore, it has been suggested to use surface treatment with chemical adhesion, micromechanical retention or a combination of both to increase bonding of PEEK to adhesives (Piwowarczyk et al., 2004). According to Caglar et al. (2018) the use of adhesive systems that contain methylmethacrylate monomer resulted in higher bond strength between PEEK and resin. The highest tensile bond strength to PEEK was achieved when the adhesive system Visio.Link (VL), which contains methylmethacrylate monomer, was combined to air‐abrasion (Stawarczyk et al., 2017).

Since there is no high‐quality evidence to support endorsing a specific type of retainer as the best retainer (Al‐Moghrabi et al., 2016); this study aimed to investigate the efficacy of PEEK wire as a fixed orthodontic retainer, by comparing its performance to other retainer wires and optimizing its adhesion to composite bonding materials.

## 2 MATERIALS AND METHODS

This study involved three tests, to measure the debonding force of retainer wires from composite, by compressive and tensile forces. A vertical compressive force was applied in two tests involving retainer wires bonded to acrylic blocks and bovine teeth, and a vertical tensile force was applied along the long axis of the wire in a pull‐out test. In all these tests, a computer‐controlled universal testing machine (Laryee WDW‐50, Beijing, China) with a 50 kN load‐cell was used. PEEK samples were milled from “White” DD‐PEEK‐MED blocks (DentalDirekt, Spenge, Germany), composed of PEEK >80%, and titanium dioxide <20% according to the manufacturer's website, using a computer numerical control (CNC) milling machine (S1, vhf camfacture AG, Ammerbuch, Germany). Three different retainer wires were used for comparison, (Re) an 0.0195‐in. dead‐soft coaxial wire (Respond, Ormco Corporation, CA), (BR) an 0.010 × 0.028‐in. three strands, stainless‐steel, braided retainer wire (OrthoTechnology Incorporation, Lutz, FL), and (FD) an 0.010 × 0.028‐in. solid flat titanium dead‐soft wire (OrthoTechnology Incorporation, Lutz, FL).

One‐hundred sixty‐three extracted bovine lower deciduous anterior teeth were obtained from freshly slaughtered calves. One‐hundred thirty‐five teeth were included in the study, that had an intact area on the lingual surface of at least 5 mm in diameter, no cracks, and no attrition affecting the lingual side.

## 3 METHODS

Cylindrical PEEK wire‐segments of 0.8 mm in diameter and 15 mm length were prepared. The dimensions of the milled pieces were verified using a digital caliper, through making perpendicular measurements in at least four different positions per a PEEK wire. All PEEK wires were finished and verified to have accurate dimensions (0.8 ± 0.03 mm).

### 3.1 Pilot study

A pilot study was conducted to determine whether light‐curing duration of VL adhesive system (BredentUK, Derbyshire, UK) has an effect on the debonding force of PEEK wires from composite bonding materials. According to the manufacturer's instructions, curing requires an ultraviolet band between 370 and 400 nm, and it takes 90 s using halogen curing units, with an emphasis that the indicator for successful polymerization is total dryness of the surface after hardening by light. A light‐emitting diode curing unit (Valo, Ultradent, Utah) was used. Eighteen PEEK wires were used and divided into three groups according to the curing time 20, 40, and 60 s, and the curing unit was used in the standard mode with 1,000 mW/cm^2^ to investigate whether curing time of VL has an effect on the debonding force. The test procedures were identical to that followed in debonding test from acrylic blocks described later. The ultimate force of failure, which is the highest force scored throughout the test, was recorded for each sample.

### 3.2 Debonding from acrylic blocks

**FIGURE 1:**
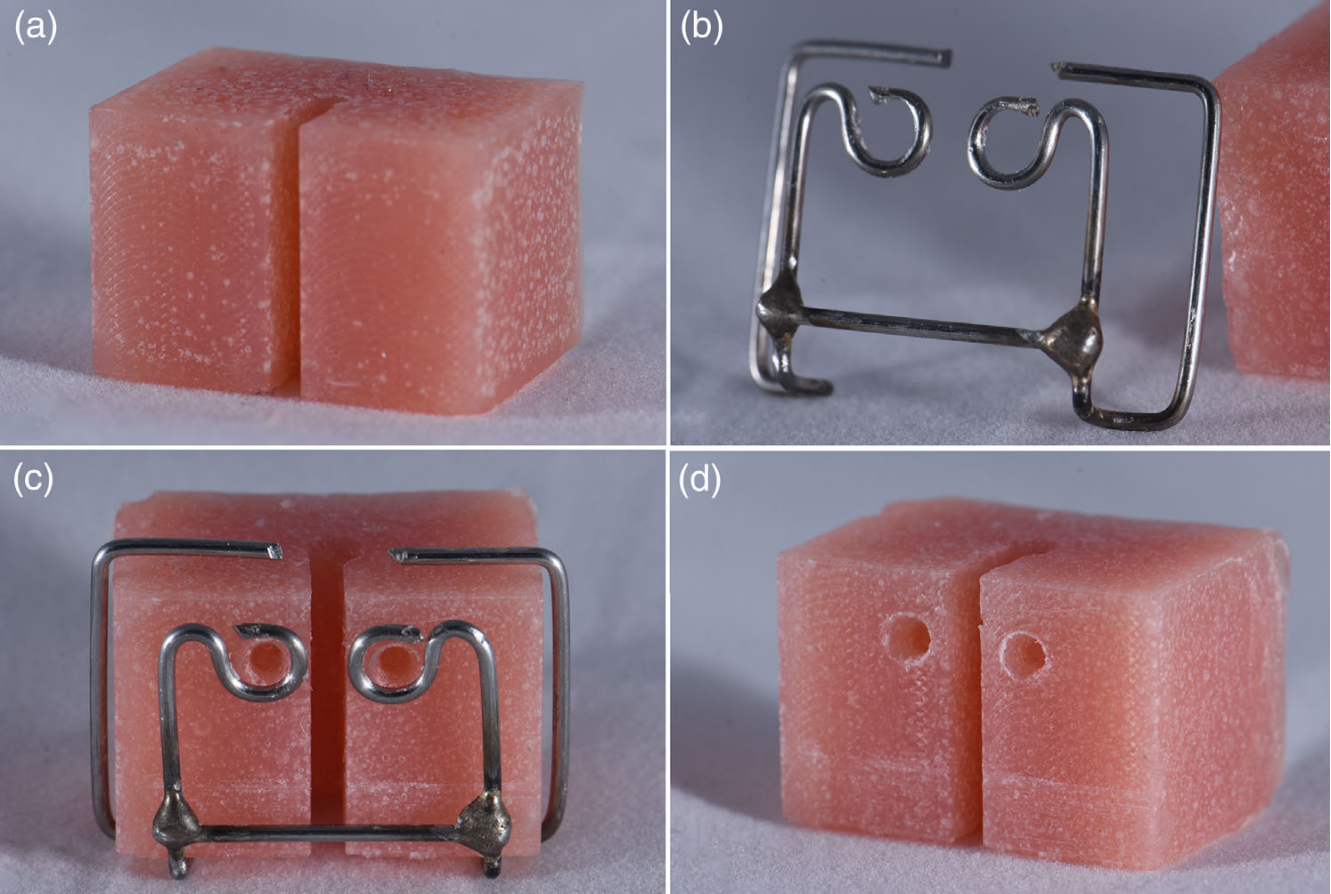
Acrylic blocks preparation. (a) Block before preparation. (b) The guide used to standardize holes position. (c) The guide in place and holes drilled. (d) Final block before bonding

A specially designed and milled brass mold was used to obtain acrylic blocks. The block consisted of two parallel arms, 7 mm in width for each, separated by a 2 mm space, to replicate the condition intraorally between the two composite bonding spots of the lower incisors. A guide made of 1 mm stainless‐steel wire was used to standardize the position of holes in the arms of the block. A large round bur (1.5 mm in diameter) was used to drill two holes of 2 mm depth through the guide. The drilled holes were cleaned off acrylic flakes and became ready for bonding (Figure 1).

Six groups of 10 lingual retainer wires were prepared (same grouping for all three tests):

- Group I (NT): PEEK wires with no surface treatment. Cleaned under running water, rinsed in 40% alcohol to remove debris, and dried.
- Group II (AA): PEEK wires that were air‐abraded for 10 s at a 10 mm distance (Supporting Information), with AL_2_O_3_ 50 μm particles at 0.35 MPa. Cleaned under running water, rinsed in 40% alcohol to remove debris, and dried.
- Group III (AV): PEEK wires that were air‐abraded for 10 s at a 10 mm distance, with AL_2_O_3_ 50 μm particles at 0.35 MPa; cleaned under running water rinsed in 40% alcohol to remove debris, dried, then conditioned with a thin layer of VL and cured for 20 s. The head of the curing unit was held as close as was possible, and curing was done against a mirror, in as close as 1 mm between the wire and the mirror, to provide curing for both sides of the wire at the same time (Supporting Information).
- Group IV (Re).
- Group V (BR).
- Group VI (FD).

Groups IV, V, and VI were cleaned under running water, rinsed in 40% alcohol to remove debris, and dried.

**FIGURE 2:**
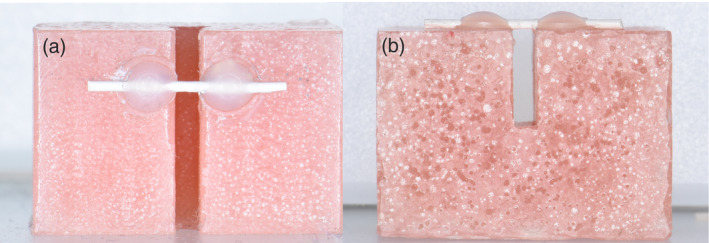
Acrylic blocks with the bonded PEEK wire. (a) Frontal view. (b) Top view

Scanning electron microscope images were taken before and after air‐abrasion of PEEK wires. The primer of Transbond XT light cure orthodontic adhesive (3M Unitek, Monrovia, CA) was applied with a brush to the hole walls and floor, the holes were then filled with adhesive. The prepared wires were placed parallel to the base of the block, with the center of the wire at the center of the blocks, and extending across the centers of the holes. To standardize the amount of composite adhesive covering the wire, a commercially available dome‐shaped wire bonder mold tips (Mini‐Mold; Ortho‐Care Ltd., Bradford, UK) were used (Supporting Information), then the composite was cured for 20 s. The prepared specimens (Figure 2) were kept in distilled water around 24 h before testing.

**FIGURE 3:**
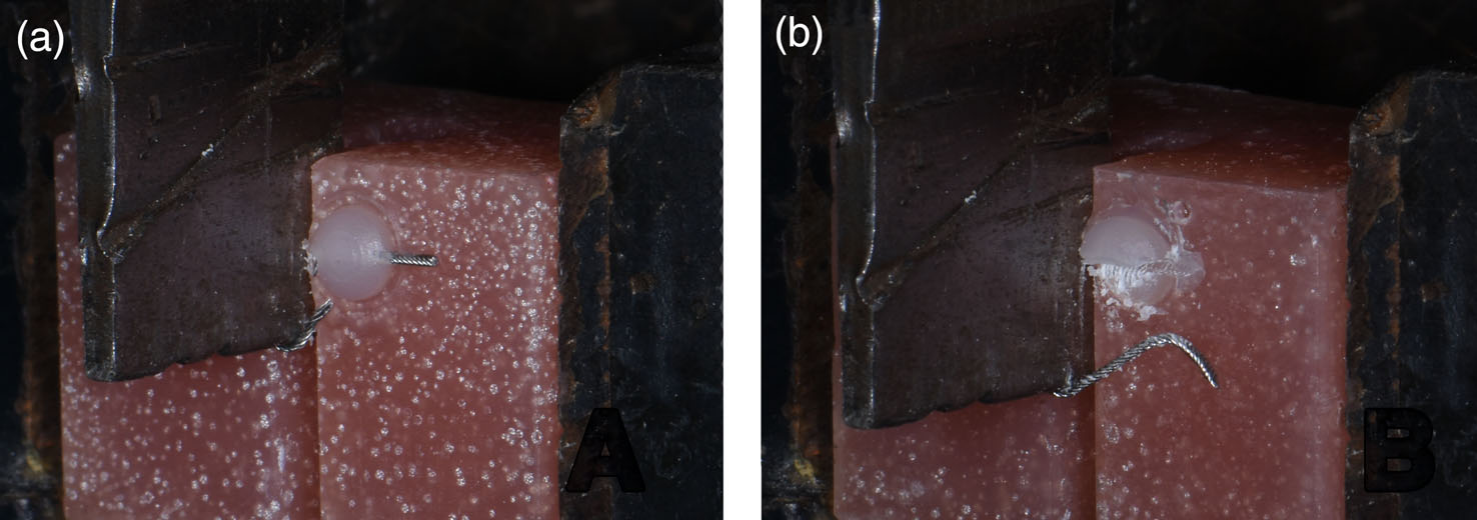
The debonding procedure was continued until the wire ruptured (a) or the composite fractured (b)

The debonding procedure was performed using a universal tension and compression machine (Laryee WDW‐50, Beijing, China) equipped with a 50 kN load cell, which had a sensitivity of 2 N. A custom‐made plunger with a round head of 1 mm in diameter was used (Supporting Information). The crosshead speed was set to 1 mm/min. The ultimate force of failure and the maximum deflection were recorded for each sample. The deflection at 30 N was selected as the maximum deflection, after which the wire often ruptured or got detached due to composite fracture; thereby the test was considered finished (Figure 3).

### 3.3 Debonding from bovine teeth

**FIGURE 4:**
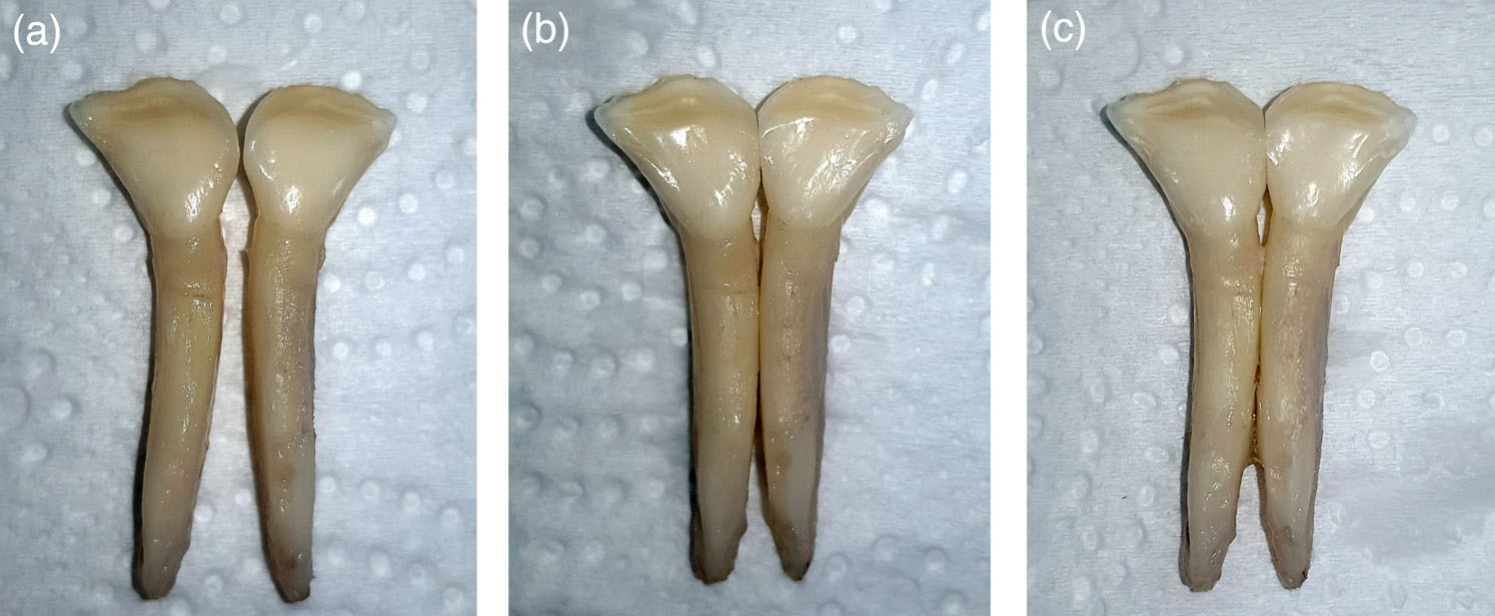
(a) Bovine teeth were trimmed from the sides (b) to provide line contact between the teeth. (c) Cyanoacrylate was used to fix the teeth in position

For debonding force from bovine teeth, the roots were cleaned with a blade and a curette to remove remnants of the periodontal tissues. The teeth were then stored in a 1% thymol solution. Since bovine incisors had a more triangular shape than human teeth, it was necessary to cut through one side of the teeth, to have intimate line‐contact between them. The trimmed teeth were initially fixed to each other using a rapid setting cyanoacrylate adhesive at the root area (Figure 4). A custom‐made T‐shaped tool was used to ensure that the lingual surfaces of the attached teeth were at the same line.

Pairs of teeth were placed in a mold and temporarily fixed in place using soft wax, making sure that the line of teeth contact was perpendicular to the block. The T‐shaped tool was attached to a surveyor and used to check that the lingual surface of the pair of teeth was well‐aligned and parallel to the side of the block (Supporting Information).

**FIGURE 5:**
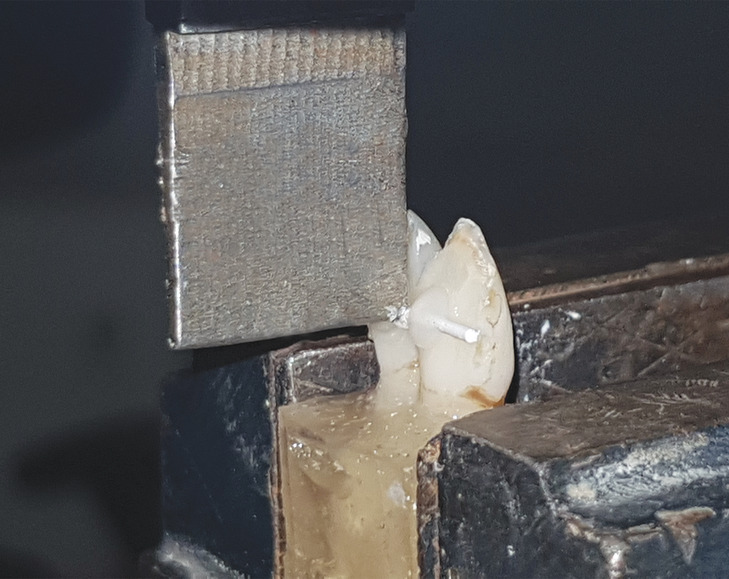
The debonding procedure on retainers bonded to bovine teeth

Cold cure acrylic was poured to form the blocks. The lingual surface of each tooth was cleaned with pumice, washed with distilled water, and subsequently dried with air. The lingual enamel surfaces were then etched with 35% orthophosphoric acid solution for 30 s (Ultra‐Etch; Ultradent Products Inc., South Jordan, Utah), washed thoroughly with water and finally dried. The primer was applied to the tooth surface without curing, the wire was bonded with the light cure adhesive on the two incisors. It was possible to provide a slight curve to the metallic wire retainers, but this was not possible with the PEEK wires as they were machined in straight pieces. The amount of adhesive was standardized with the use of molds, then cured for 20 s. The test samples were kept in distilled water for 24 h before running the test. The debonding procedure was identical to what has been described previously, and maximum deflection before final failure was also recorded at 30 N (Figure 5).

### 3.4 Pull‐out test

**FIGURE 6:**
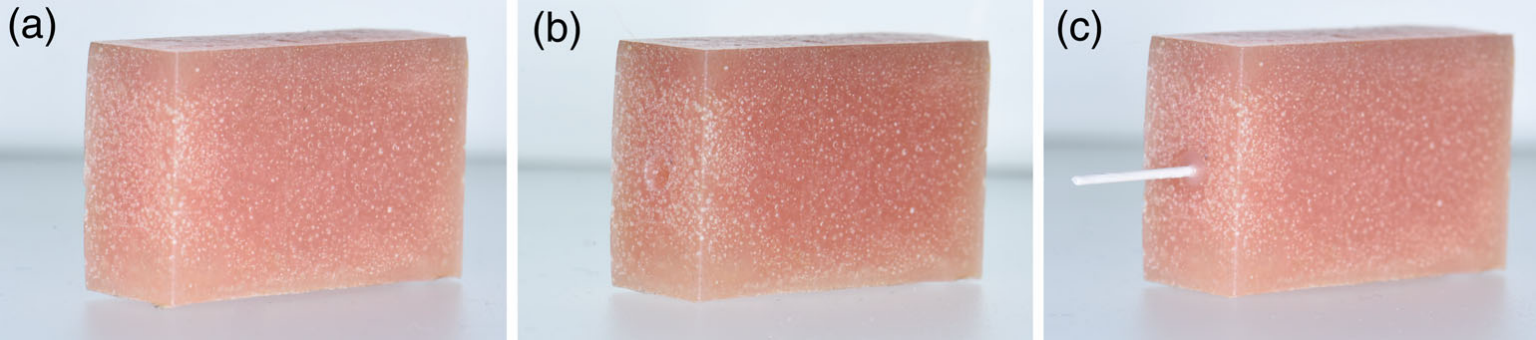
The block used in the pull‐out test (a), a hole drilled in the center of the block (b), with the retainer wire bonded (c)

To perform pull out test, blocks were prepared of acrylic with a hole of 2 mm in width, and 3 mm in depth, drilled at the center of an acrylic block. The hole was cleaned off remnants of acrylic. The prepared wire was placed parallel to the long axis of the block, at the center of the holes, and checked with a surveyor from all sides. The adhesive was cured for 20 s. The test samples were kept in distilled water for around 24 h before running the test (Figure 6).

**FIGURE 7:**
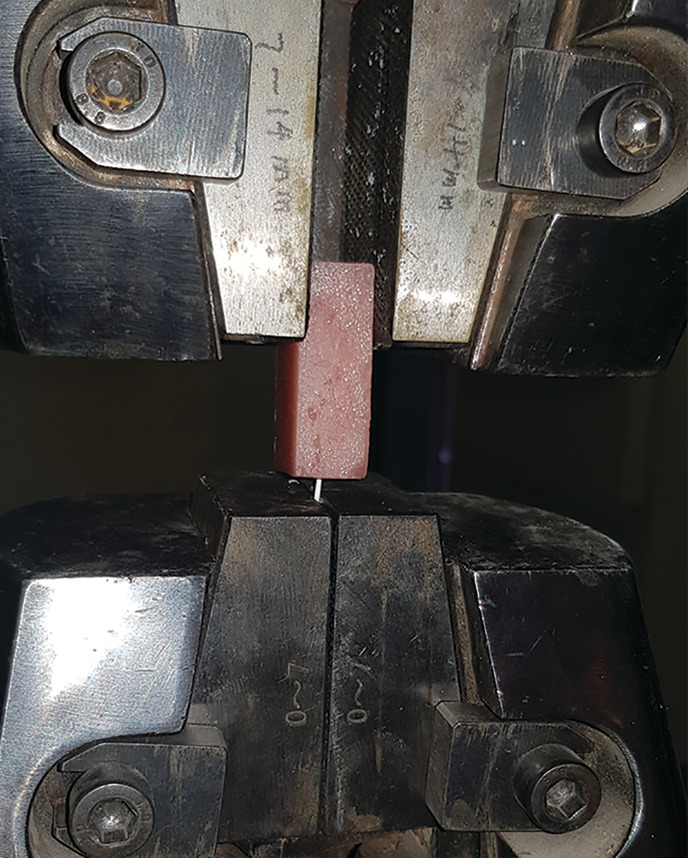
The test block is attached to the movable upper jaw, while the wire is firmly grabbed by the lower fixed jaw

The block and wire were aligned so that the pull was along the long axis of the block (Figure 7). The crosshead speed was set to 10 mm/min. The ultimate force of failure was recorded for each sample as the highest force recorded during a pull‐out test. The test was continued until either the wire failed by rupturing, or got detached from composite.

## 4 STATISTICAL ANALYSIS

Statistical analyses were performed using the SPSS 26.0 (SPSS Inc., Chicago, IL). The Shapiro–Wilks normality test and Levene's variance homogeneity test were applied to the data. For normally distributed data with equal variances, one‐way ANOVA and Tukey HSD post hoc multi‐comparison test were used. While those with unequal variances Welch F test and Games‐Howell post hoc multi‐comparison test were used.

## 5 RESULTS

All data were normally distributed, with unequal variances with the exception of the ultimate force of failure of retainers bonded to bovine teeth. The *p*‐value ≤0.05 was considered significant; however, as a result of multiple testing—measuring force and deflection at the same time—Bonferroni correction was applied with a *p*‐value ≤0.025.

### 5.1 Scanning electron microscope imaging

Scanning electron microscope images taken before air‐abrasion showed a distinctive pattern on the wire surface, which was attributed to the cutting burs of the CNC machine. The surface was relatively uniform with some elevations and depressions. The images taken after air‐abrasion showed total loss of that pattern, with deep grooves and highly irregular surface (Supporting Information).

### 5.2 Pilot study

Curing VL for 20 s was sufficient to develop a dry surface. No significant difference was found between the three groups; so, it was decided to use the 20 s curing light time for VL in the remaining tests of this study (Table 1 and Supporting Information).

**TABLE 1:** Descriptive statistics of the effect of different light curing time of VL on the ultimate force of failure

	*N*	Mean	*SD*	Minimum	Maximum	Significance*
20s	6	86.50	5.05	82.00	96.00	Non‐significant difference
40s	6	82.17	5.74	72.00	88.00	
60s	6	88.83	12.48	75.00	105.00	

^*^ The *p*‐value is significant at the 0.05 level.

### 5.3 Debonding from acrylic blocks

**FIGURE 8:**
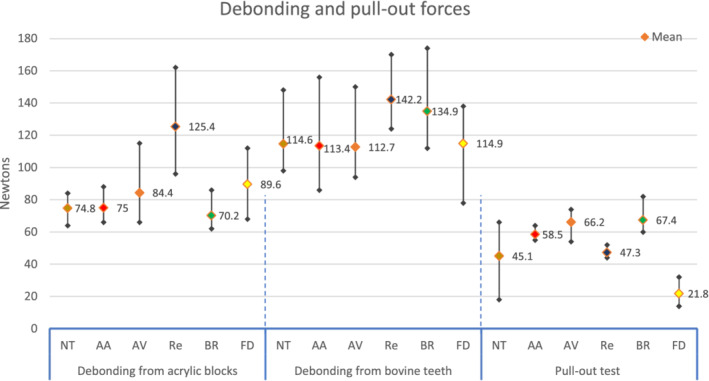
The ultimate force of failure of debonding and pull out tests

**FIGURE 9:**
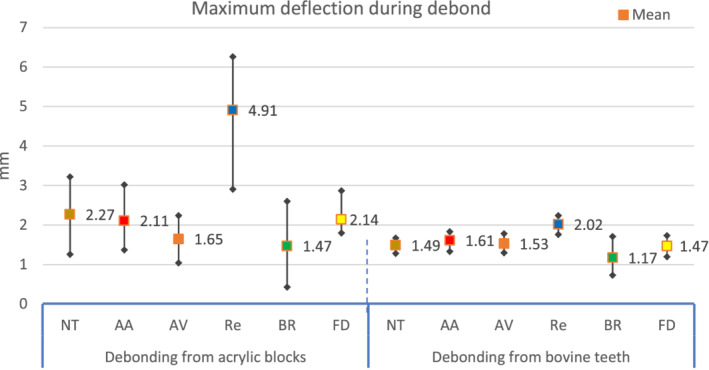
The maximum deflection of debonding tests

**FIGURE 10:**
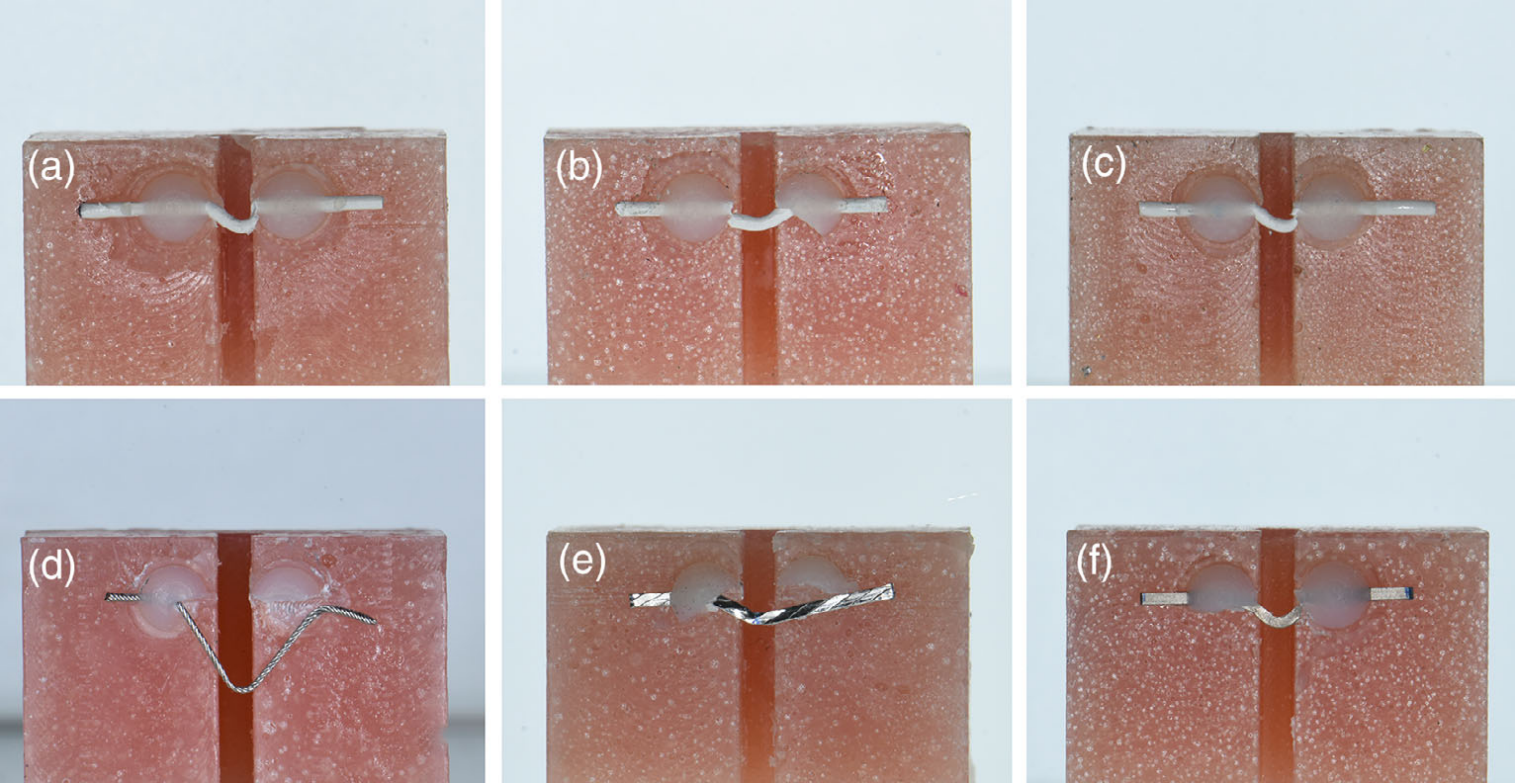
Debonding force measurement performed on retainer wires bonded to acrylic blocks. PEEK wires (a) no treatment, (b) air‐abraded, (c) air‐abraded with Visio.Link. Metal wires (d) Respond, (e) Braided‐retainer, (f) Flat dead titanium

There was a statistically significant difference between groups (Table 2), post hoc multi comparisons showed a significant difference between the ultimate force of failure of Re as compared to other retainer wires. PEEK wires (regardless the surface treatment) had non‐significant difference when compared to each other, or to the other two metallic wires. The BR had a significant difference when compared to FD. The highest ultimate force of failure of Re was associated with the greatest deformation of the wire before failure (Figures 8, 9, 10). When the maximum deflection data was inspected, there was a significant difference between groups, that was between the maximum deflection of Re and all other wires (Table 3).

**TABLE 2:** Descriptive and inferential statistics of the ultimate force of failure of debonding and pull out tests

Test	Retainer wire groups	*N*	Mean	*SD*	Min	Max	Significance*
Debonding from acrylic blocks	NT	10	74.80	6.39	64	84	Re*
	AA	10	75.00	7.12	66	88	Re*
	AV	10	84.40	14.52	66	115	Re*
	Re	10	125.40	21.71	96	162	NT, AA, AV, BR, FD*
	BR	10	70.20	7.02	62	86	Re, FD*
	FD	10	89.60	14.14	68	112	Re, BR*
Debonding from bovine teeth	NT	10	114.60	14.56	98	148	Re*
	AA	10	113.40	18.21	86	156	Re*
	AV	10	112.70	17.66	94	150	Re*
	Re	10	142.20	16.07	124	170	NT, AA, AV, FD*
	BR	10	134.90	18.02	112	174	Non‐significant*
	FD	10	114.90	16.81	78	138	Re*
Pull‐out test	NT	10	45.10	16.83	18	66	AV, BR, FD**
	AA	10	58.50	2.84	55	64	AV, Re, BR, FD**
	AV	10	66.20	6.14	54	74	AA, Re, FD**
	Re	10	47.30	2.45	44	52	AA, AV, BR, FD**
	BR	10	67.40	6.26	60	82	NT, AA, Re, FD**
	FD	10	21.80	5.43	14	32	NT, AA, AV, Re, BR**

*Note:* All measurements are in Newtons.

^*^ The *p*‐value is significant at the 0.025 level.

^**^ The *p*‐value is significant at the 0.05 level.

**TABLE 3:** Descriptive and inferential statistics of the maximum deflection of debonding tests

Test	Retainer wire groups	*N*	Mean	*SD*	Min	Max	Significance
Debonding from acrylic blocks	NT	10	2.27	0.79	1.26	3.22	Re*
	AA	10	2.11	0.58	1.37	3.02	Re*
	AV	10	1.65	0.31	1.04	2.24	Re*
	Re	10	4.91	1.03	2.91	6.26	NT, AA, AV, BR, FD*
	BR	10	1.47	0.61	0.43	2.60	Re*
	FD	10	2.14	0.33	1.80	2.87	Re*
Debonding from bovine teeth	NT	10	1.49	0.12	1.28	1.67	Re*
	AA	10	1.61	0.17	1.33	1.83	Re, BR*
	AV	10	1.53	0.15	1.30	1.78	Re*
	Re	10	2.02	0.17	1.76	2.24	NT, AA, AV, BR, FD*
	BR	10	1.17	0.32	0.73	1.71	AA, Re*
	FD	10	1.47	0.14	1.20	1.73	Re*

*Note:* All measurements are in mm.

^*^ The *p*‐value is significant at the 0.025 level.

### 5.4 Debonding from bovine teeth

**FIGURE 11:**
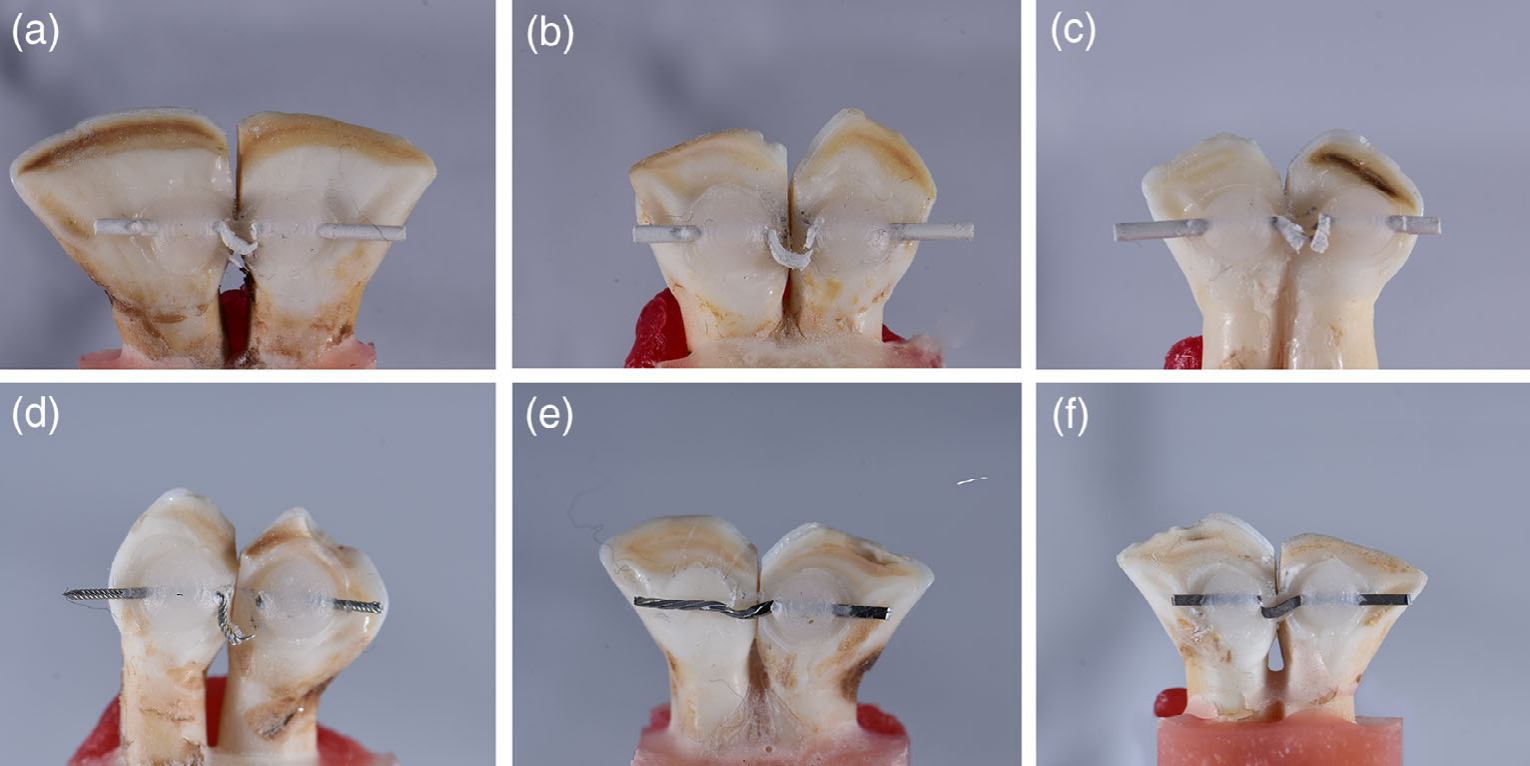
Debonding force measurement performed on retainer wires bonded to bovine teeth. PEEK wires (a) no treatment, (b) air‐abraded, (c) air‐abraded with Visio.Link. Metal wires (d) Respond, (e) Braided‐retainer, (f) Flat dead titanium

The ultimate force of failure differed significantly between the different groups. The Re group had significantly higher ultimate force of failure than all other groups, except BR. There was a non‐significant difference among all other groups. All retainer‐wire failure was by wire rupture, except BR, where the failure was by composite fracture (Figure 11). The Re group also had the greatest maximum deflection, which was significantly different when compared to all other groups. Only AA had a significant difference when compared to BR, all other groups showed no significant differences.

### 5.5 Pull‐out test

**FIGURE 12:**
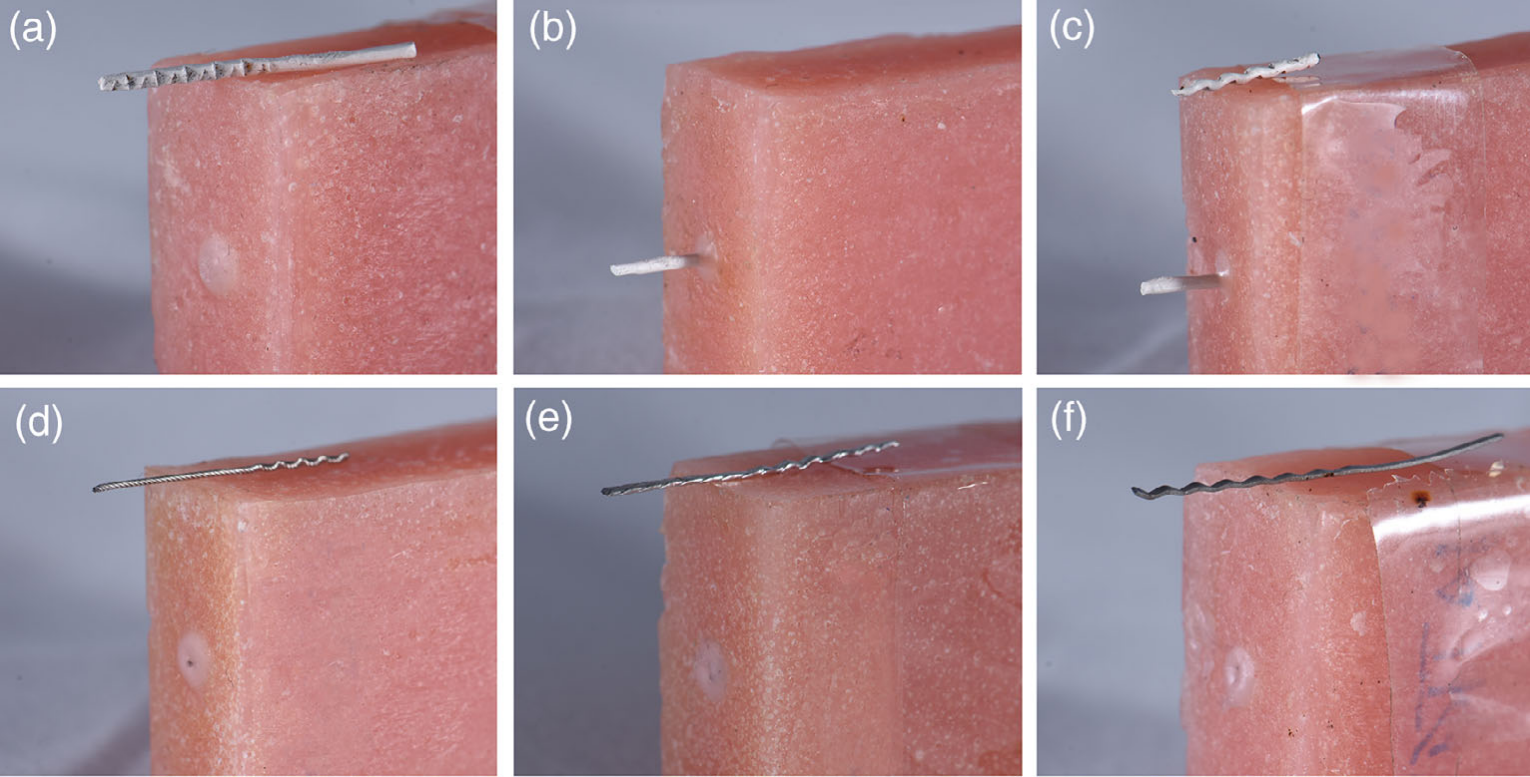
During the pull‐out test, only the air‐abraded and air abraded with Visio.Link PEEK wires rupture, the other wires got detached from the block. PEEK wires (a) no treatment, (b) air‐abraded, (c) air‐abraded with Visio.Link. Metal wires (d) Respond, (e) Braided‐retainer, (f) Flat dead titanium

There was a statistically significant difference when the pull‐out force data was inspected. For the PEEK groups, AV had a statistically significant higher pull‐out force when compared to NT and AA. The FD group had a significantly lower ultimate force of failure when compared to all groups. During the test, the AA and AV groups had the wire ruptured during the test; while the wire came out of the block for all other groups (Figure 12).

## 6 DISCUSSION

Most patients would require a fixed lingual retainer to keep their anterior teeth stable after orthodontic treatment (Renkema et al., 2008). The PEEK is known for its remarkable mechanical properties. It can be modified with additives, like carbon fibers‐reinforced, hydroxyapatite, barium sulfate, and titanium dioxide, to change its biomechanical properties and behavior (Kurtz, 2019). PEEK compounds, with and without additives, have the good fatigue resistance properties to withstand cyclic loading. Titanium dioxide increases the stiffness of PEEK, in addition to its original use as a whitening agent (Schwitalla et al., 2017). It was shown that PEEK with 20% TiO_2_ filler particles had the highest bond strength when compared to a higher or a lower filler content (Lümkemann et al., 2017).

The mechanical behavior of the wires was investigated by performing three tests. The first test was intended to measure the force required to break the bond between the wire and the composite adhesive through the application of a compressive force. Fixing the wire to the U‐shaped acrylic blocks with the composite resin secured to a hole within the block, ensured eliminating bonding to enamel as a variable; also having the plunger passing freely in the slot of the block without any interference, reflected the actual bonding force. The second test was adopted from the works of Cooke and Sherriff (2010) and Baysal et al. (2012), who investigated the combined effect of bonding of the adhesive to the wire and the bond to enamel. The third test involved measuring bonding strength while the retainer wire is subjected to a tensile force, it was adopted from the works of Bearn et al. (1997) and Baysal et al. (2012).

Surface treatment is necessary to increase bonding to PEEK as it is an inert material. Air‐abrasion results in roughening of the surface, which will aid in a greater surface area and more interlocking between the PEEK and the adhesive. Moreover, with VL, the presence of pentaerythritol triacrylate modifies the PEEK surface and could result in better bonding with the resin adhesives (Caglar et al., 2018). Since there has been no previous research on bonding to PEEK in the wire form, it was decided to investigate the effects of both surface treatments on debonding force as compared to the non‐treated sample.

The performance of PEEK was compared to three retainer wires; a commonly used dead‐soft six strand coaxial stainless‐steel wire, a braided stainless‐steel retainer wire with a flat profile that helps retaining the final torque positions and reduce occlusal interference, and a third single‐strand titanium wire, that has, in addition to the features of the braided stainless steel wire, a reduced wear rate and is nickel‐free.

## 7 PILOT STUDY

According to the manufacturer's instruction, curing VL requires an ultraviolet band between 370 and 400 nm. Valo LED curing units cover a range of 385–515 nm according to the official website of the product (Ultradent, 2017). There was no data regarding the required time for curing VL using Valo, and whether that time could affect the strength of bonding to PEEK; consequently, a pilot study was performed.

The results of the pilot study showed that there is a non‐significant difference between the three light‐curing times, 20, 40, or 60 s. Hence, it was decided to use the 20 s as the light‐curing time of VL for the remaining tests of this study.

### 7.1 The debonding force of retainer‐wires bonded to acrylic blocks

The NT and AA groups had very close results. The results of AV, on the other hand, were greater in the magnitude of the ultimate force of failure, and smaller in the maximum deflection before failure, which could reflect a more rigid behavior; however, these differences were not statistically significant. The pattern of failure for AA and AV groups was due to wire rupture, with minor composite fractures. While the NT group had 2 out of 10 samples where composite was fractured and the wire became free, indicating a greater stress build‐up in the composite. The FDT group had failures due to wire rupture, with only 5 out of 10 samples have partial composite fracture. On the contrary, none of the BR group had wire rupture, and failure was due to composite fracture. Finally, the Re group had composite fracture as a cause of failure, with a single wire rupture. The Re group had the highest ultimate force of failure which peaked out among other groups, and it had the greatest maximum deflection before failure. Both can be attributed to its high ductility.

There is no research with a similar laboratory set up to provide a direct comparison of the results. However, Re wire groups were associated with a higher ultimate force of failure and maximum deflection before failure (Milheiro et al., 2012).

The more deformation before a failure happens, the more the possibility that the teeth are moved to a new position without having the wire ruptured or composite fractured, this is mostly the case with Re group.

### 7.2 The debonding force of retainer‐wires bonded to bovine teeth

Several non‐human teeth have been used in research, these include swine, equine, bovine, and shark teeth (Yassen et al., 2011). Bovine lower incisors are much easier to collect and are an inexpensive substitute to human incisors, do not have carious lesions or other defects that may affect test outcomes (Oesterle et al., 1998; Yassen et al., 2011). In their systematic review and meta‐analysis, de Carvalho et al. (2018) concluded that bovine teeth are a viable alternative to human teeth.

The results of the ultimate force of failure of retainer wires were greater than their counterparts on acrylic blocks. The vertically applied force would have two reactive components, vertical and horizontal, arising from contacting the inclined tooth surface. It was previously confirmed that the horizontal component of force was greater than the vertical component (Sifakakis et al., 2011). These would resist the downward deflection of the wire and contribute to greater force measurement.

The PEEK wires, regardless of the surface treatment, had comparable performance to the other retainer wires, with no statistically significant difference, except to Re group. They all have ruptured before the composite got released from composite. The differences in maximum wire deflection to other wires are not statistically, nor clinically significant. The Re group recorded much greater force when compared to the results of Baysal et al. (2012), this is mostly attributed to the larger gap between the bonding spots which was used in that research.

### 7.3 The pull‐out test

The pull‐out test helped to further unveil the differences among the retainer groups. It is known that the greater the surface area the greater the force required to remove (Bearn et al., 1997). The FD group had a significantly lower force of failure as compared to all other retainer groups. This can be mainly attributed to the fact that it is a flat and single‐stranded wire; therefore, its surface area is smaller than the closest match, the BR group, which despite having the same dimensions, but being made of three strands of annealed stainless‐steel wires has added to the surface area, and provided more gaps for the resin adhesive to bond. The Re and NT groups were in the lower rank when the pull‐out force is concerned, with a non‐significant difference between them. Even though the Re group had a smaller diameter, but its multistrand nature would add to the surface area and contribute to the retention of adhesive to the wire. The other PEEK groups namely AA and AV had greater pull‐pull out force, this again highlights the importance of the surface area, because air‐abrasion resulted in greater surface irregularities for the resin composite to lock‐in, and the effect of the adhesive system VL seemed to increase the bonding force. However, it seemed that air‐abrasion scratches may result in some weakening of the wire, therefore both groups have ruptured at slightly higher pull‐out forces, while the NT group came out intact. Even though the increase in pull out force between the AV and AA groups is statistically significant, it seems of little clinical significance, especially when we consider the failure pattern for both which is wire rupture.

### 7.4 Limitations of the study

This study has certain limitations such as; lacking a specific protocol to cutting straight pieces of PEEK in a small round cross‐section. The protocol of cutting PEEK for crown and bridge work was modified to get the retainer wire pieces. The manual finishing of the PEEK wires, although it is believed that the effect of this variable is limited by keeping the variation within 0.03 mm after finishing. Other limitations include the use of bovine instead of human teeth and the variable morphology and size of the bovine teeth. Natural teeth tend to have various morphologies as well, so this was considered a better representation of the in vivo situation. Besides, standardizing the amount of bonding material and keeping fixed distance between composite bonding spots would minimize the effect of this variability.

## 8 STRENGTH OF THE STUDY

The strength of the study can be seen in that it is the first study which investigates the performance of PEEK wires with different surface treatments as a fixed retainer wire and compares its performance to other metallic counterparts.

## 9 CONCLUSIONS

Within the limitations of this in‐vitro study regarding the efficacy of PEEK wire as a retainer after orthodontic treatment, it can be concluded that the 0.8 mm round wire‐form has comparable performance—in terms of debonding and pull out forces—to conventional retainers when bonded with 4 mm composite bonding spots; using air‐abrasion for 10 s at 3.5 MPa provided adequate adhesion of the composite to the wire, and conditioning with the adhesive system Visio.Link may provide no further clinical benefit.

The use of PEEK wire over other metallic wires is supported for future in vivo study to confirm these outcomes.

## AUTHOR CONTRIBUTION

ASK contributed to the design, conducted the study, collected data, performed analysis, interpreted the results, and wrote the manuscript.

AFA designed the study, and helped interpreting the results and preparing the manuscript.

## CONFLICT OF INTEREST

The authors have nothing to disclose.

## CLINICAL SIGNIFICANCE

The stable PEEK structure with good bonding to composite (after simple preparation), could aid in controlling the labiolingual, mesiodistal, incisogingival, and rotation along the long axis, which would most likely reduce post‐retention changes seen with other retainers.

## Supporting information


**Appendix** **S1:** Supporting information

## Data Availability

Data available on request from the authors.
