# Supplementary material for: The efficacy of polyether‐ether‐ketone wire as a retainer following orthodontic treatment
Source: Clin Exp Dent Res. 2020 Dec 13;7(3):302–12. doi: 10.1002/cre2.377 (PMC8204027; doi:10.1002/cre2.377)
Supplement: Supplementary file 1 — Appendix S1: Supporting information [file CRE2-7-302-s001.zip › CRE2_377_cre2.20200291-File014.docx]

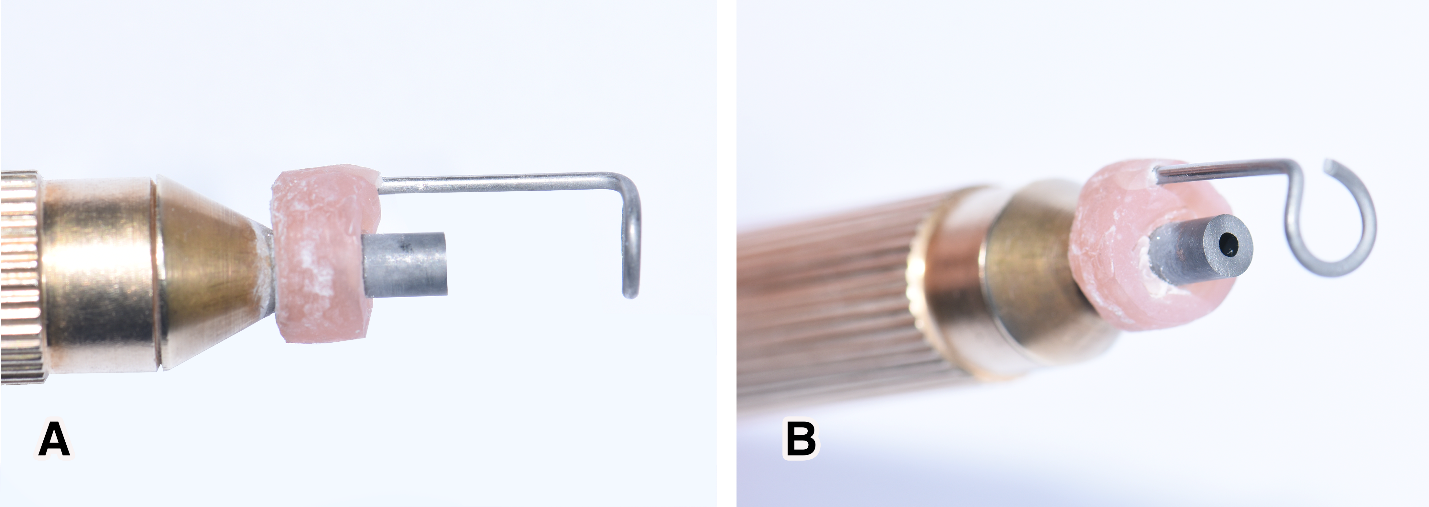


A custom-made tip was fabricated and fitted to the head of the air-abrading machine, to standardize ‎the distance at ‎‎10mm.‎
