# Supplementary material for: The efficacy of polyether‐ether‐ketone wire as a retainer following orthodontic treatment
Source: Clin Exp Dent Res. 2020 Dec 13;7(3):302–12. doi: 10.1002/cre2.377 (PMC8204027; doi:10.1002/cre2.377)
Supplement: Supplementary file 1 — Appendix S1: Supporting information [file CRE2-7-302-s001.zip › CRE2_377_cre2.20200291-File020.docx]

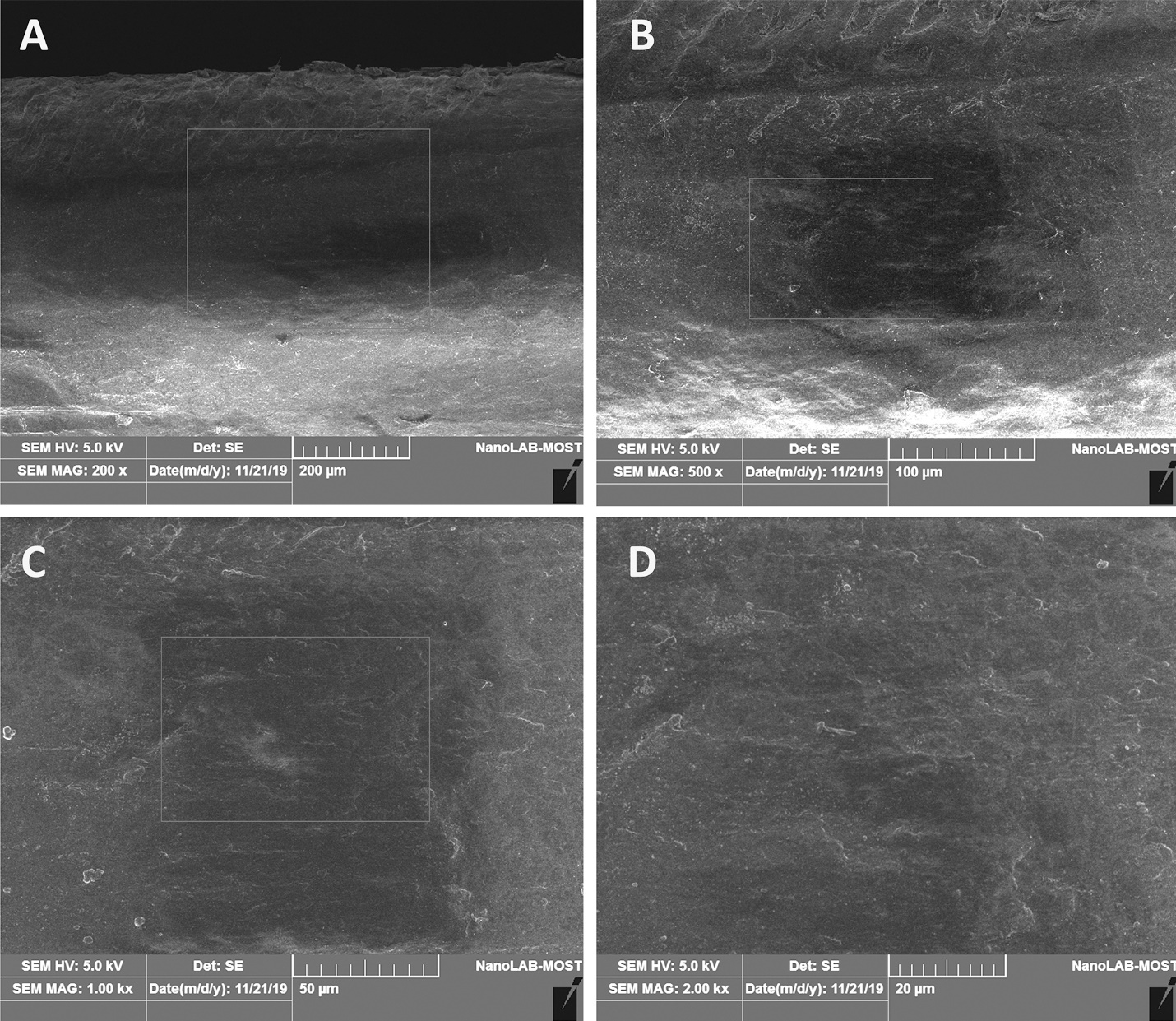


Scanning electron microscope before air-abrasion with different magnification settings, A, 200X, B, 500X, C, 1000X, and D, 2000X.
