# Supplementary figures and images for: The efficacy of polyether‐ether‐ketone wire as a retainer following orthodontic treatment
Source: Clin Exp Dent Res. 2020 Dec 13;7(3):302–12. doi: 10.1002/cre2.377 (PMC8204027; doi:10.1002/cre2.377)

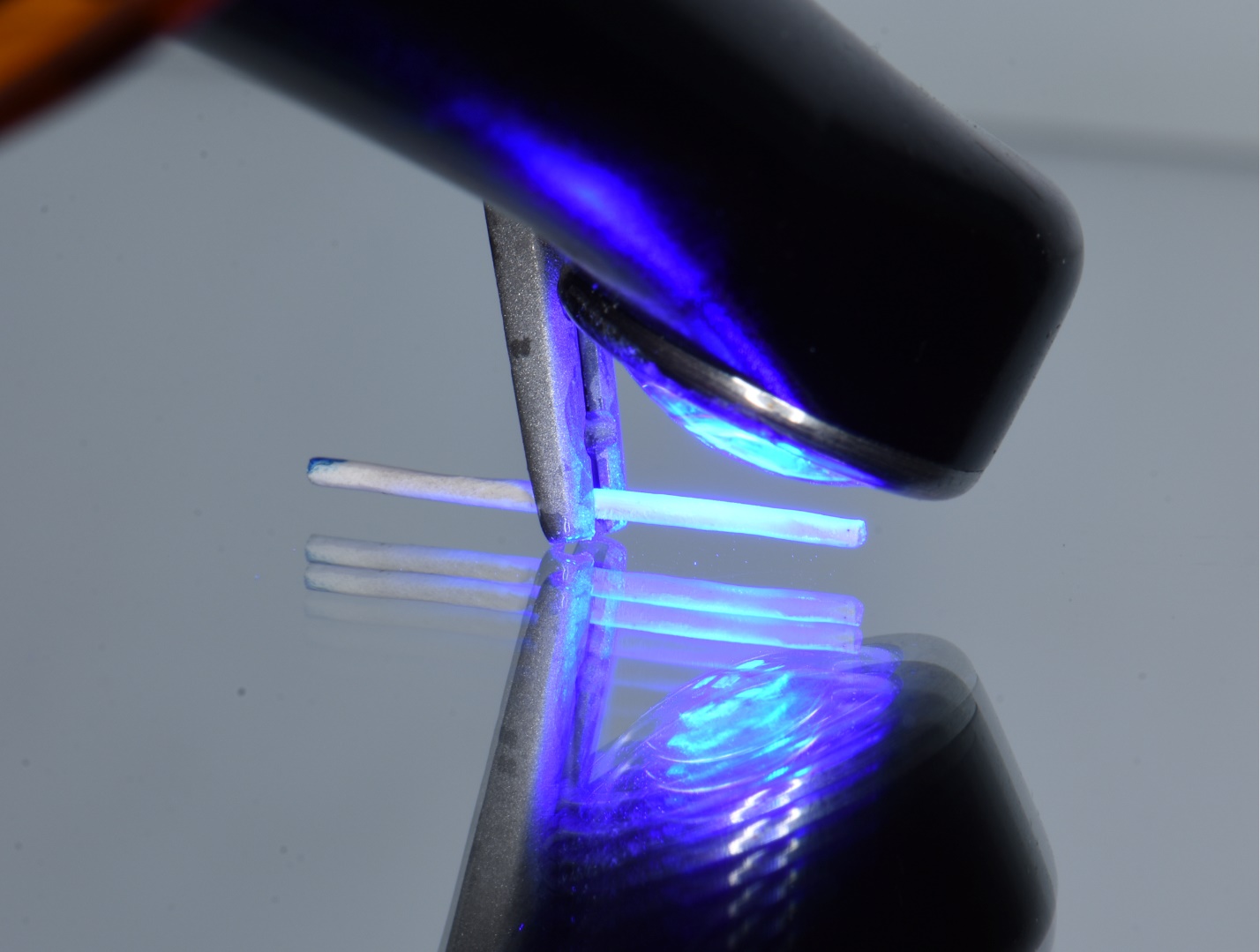


Curing VL was done against a mirror.

Supplement: Supplementary file 1 — Appendix S1: Supporting information [file CRE2-7-302-s001.zip › CRE2_377_cre2.20200291-File015.docx]

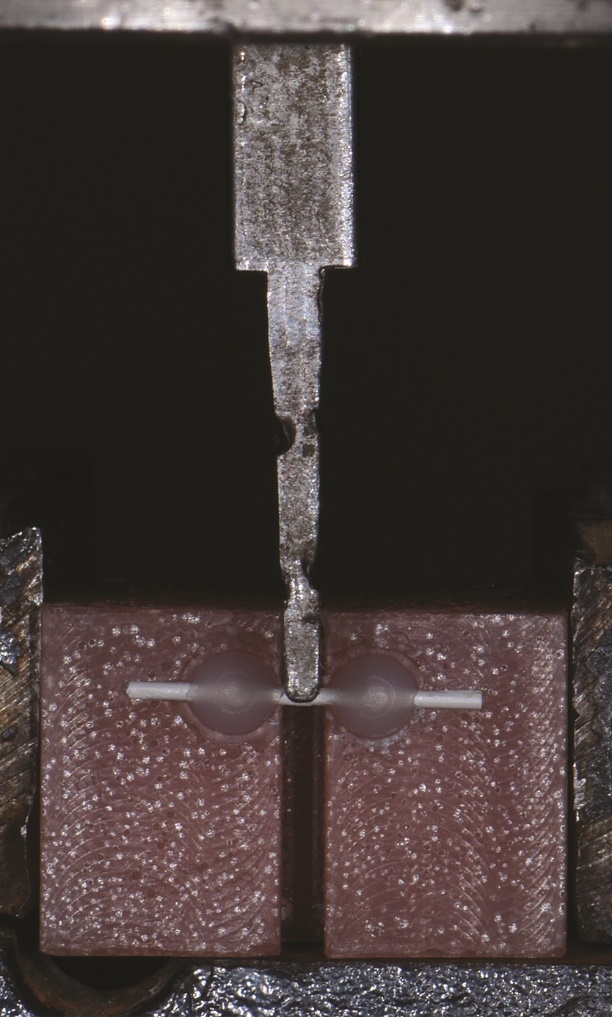


Test blocks in place during the debonding procedure

Supplement: Supplementary file 1 — Appendix S1: Supporting information [file CRE2-7-302-s001.zip › CRE2_377_cre2.20200291-File017.docx]

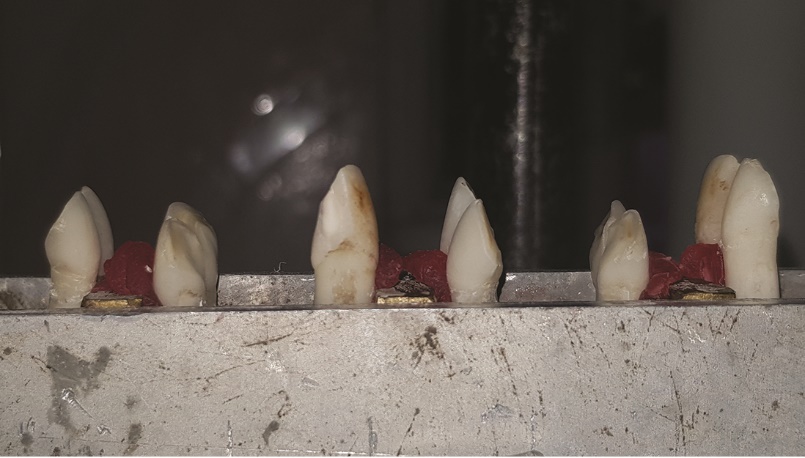


The teeth were temporarily fixed with wax before pouring the acrylic.

Supplement: Supplementary file 1 — Appendix S1: Supporting information [file CRE2-7-302-s001.zip › CRE2_377_cre2.20200291-File018.docx]

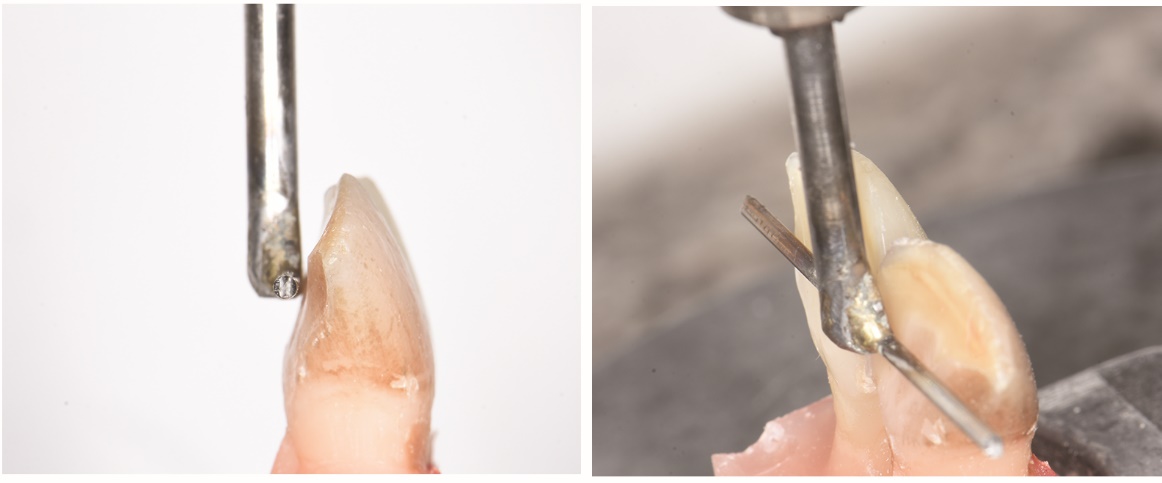


A T-shaped tool was used to make sure the lingual surface is in good alignment.

Supplement: Supplementary file 1 — Appendix S1: Supporting information [file CRE2-7-302-s001.zip › CRE2_377_cre2.20200291-File019.docx]

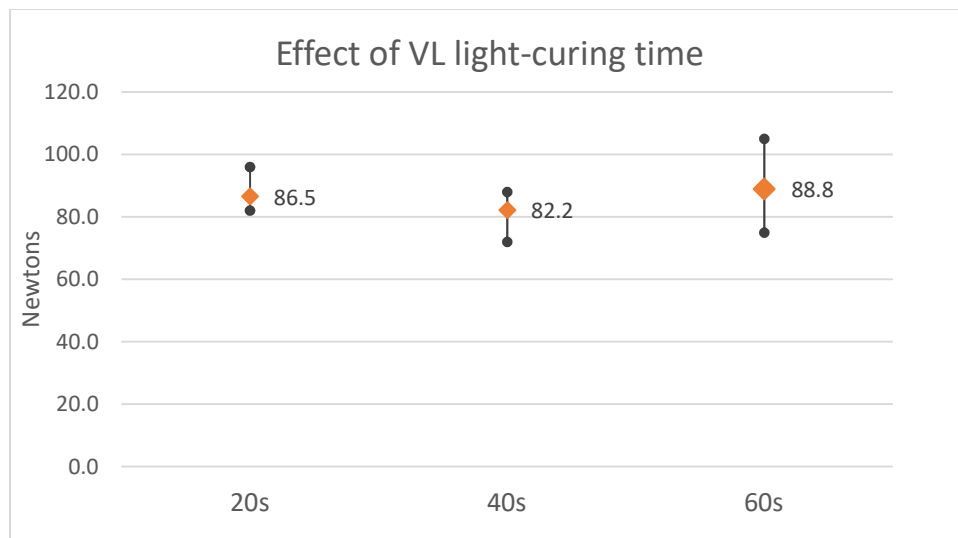

Effect of three light-curing time on the maximum failure force in debonding

Supplement: Supplementary file 1 — Appendix S1: Supporting information [file CRE2-7-302-s001.zip › CRE2_377_cre2.20200291-File022.pdf]
